# From Molecules to Medicines: The Role of AI-Driven Drug Discovery Against Alzheimer’s Disease and Other Neurological Disorders

**DOI:** 10.3390/ph18071041

**Published:** 2025-07-14

**Authors:** Mashael A. Alghamdi

**Affiliations:** Department of Chemistry, College of Science, Imam Mohammad Ibn Saud Islamic University (IMSIU), Riyadh 11432, Saudi Arabia; mabalghamdi@imamu.edu.sa

**Keywords:** machine learning, artificial intelligence, proteomics, Alzheimer’s disease, drug discovery, genomics

## Abstract

The discovery of effective therapeutics against Alzheimer’s disease (AD) and other neurological disorders remains a significant challenge. Artificial intelligence (AI) tools are of considerable interest in modern drug discovery processes and, by exploiting machine learning (ML) algorithms and deep learning (DL) tools, as well as data analytics, can expedite the identification of new drug targets and potential lead molecules. The current study was aimed at assessing the role of AI-based tools in the discovery of new drug targets against AD and other related neurodegenerative diseases and their efficacy in the discovery of new drugs against these diseases. AD represents a multifactorial neurological disease with limited therapeutics available for management and limited efficacy. The discovery of more effective medications is limited by the complicated pathophysiology of the disease, involving amyloid beta (Aβ), neurofibrillary tangles (NFTs), oxidative stress, and inflammation-induced damage in the brain. The integration of AI tools into the traditional drug discovery process against AD can help to find more effective, safe, highly potent compounds, identify new targets of the disease, and help in the optimization of lead molecules. A detailed literature review was performed to gather evidence regarding the most recent AI tools for drug discovery against AD, Parkinson’s disease (PD), multiple sclerosis (MLS), and epilepsy, focusing on biological markers, early diagnoses, and drug discovery using various databases like PubMed, Web of Science, Google Scholar, Scopus, and ScienceDirect to collect relevant literature. We evaluated the role of AI in analyzing multifaceted biological data and the properties of potential drug candidates and in streamlining the design of clinical trials. By exploring the intersection of AI and neuroscience, this review focused on providing insights into the future of AD treatment and the potential of AI to revolutionize the field of drug discovery. Our findings conclude that AI-based tools are not only cost-effective, but the success rate is extremely high compared to traditional drug discovery methods in identifying new therapeutic targets and in the screening of the majority of molecules for clinical trial purposes.

## 1. Introduction

The discovery of new drugs is an extremely multifaceted, painstaking, and costly process that consists of identifying potential therapeutic targets, designing and synthesizing molecules, and testing their effectiveness and safety in preclinical and clinical trials [1]. Due to multiple and time-consuming processes, drug discovery for many diseases, including infectious diseases and neurological disorders, is extremely slow [2]. The random selection of target molecules and screening through various preclinical and clinical phases are not only costly, but the success rate of molecules as potential leads is also low [3]. Random screening is not only costly but may also be associated with toxic responses in patients, and the subsequent withdrawal from clinical use is too costly for researchers, the pharmaceutical industry, and patients. More importantly, the drug development process takes too long to make drugs available for patient use [4,5]. With the recent developments in the scientific and technological aspects of drug discovery, AI has appeared as a powerful tool, allowing investigators to scrutinize huge quantities of data, identify patterns, and predict the roles of new molecules [6]. By leveraging AI tools like ML and DL, researchers can identify potential drug candidates more efficiently, optimize their properties, and predict their behavior in the body [7].

AI tools are effectively utilized during various steps of drug discovery, like the identification of drug targets, designing compounds capable of interacting with targets, virtual screening, and the prediction of the pharmacokinetic and pharmacodynamic aspects of potential target molecules [8]. For example, AI algorithms effectively analyze large datasets for the identification of chemical structures and their biological potential, which helps to recognize new drug targets, and they are capable of predicting the safety and efficacy of possible drug molecules [9]. Other extremely important features of AI use include information about drug absorption, distribution, metabolism, and elimination (pharmacokinetics), which indicates the drug’s bioavailability, as well as the interaction of the drug molecules at the target site proteins/receptors (pharmacodynamics) [10]. All of these processes for random molecules are highly time-consuming and costly, and the chance of a usable drug is also quite low, whereas the assimilation of AI tools has the capacity to transform the discovery and development of novel therapeutic agents and help the pharmaceutical industry by decreasing the required time, costs, and risks associated with introducing new medicines to the marketplace [11]. Keeping in mind the importance of AI tools in drug discovery, it has been reported that the role of AI in the global healthcare market may increase from 2.1 billion (2020) to 31.3 billion (2030) as a vital driver in drug discovery [12,13].

This review was aimed at providing a comprehensive overview of the potential role of AI-based tools in the discovery and development of novel therapeutic moieties against AD and other related neurological disorders, including Parkinson’s disease (PD), multiple sclerosis (MS), epilepsy, and amyotrophic lateral sclerosis (ALS). This work focused on the applications of the most recent AI tools in drug discovery, highlighting recent success stories and the identification of novel therapeutic targets; the design of effective small molecules; and predictions of their safety, efficacy, and potential toxicities. By exploring the intersection of AI and neurodegenerative diseases, this review seeks to deliver a nuanced understanding of the potential benefits and limitations of AI-based drug discovery approaches.

## 2. Traditional Processes of Drug Discovery

**Figure 1:**
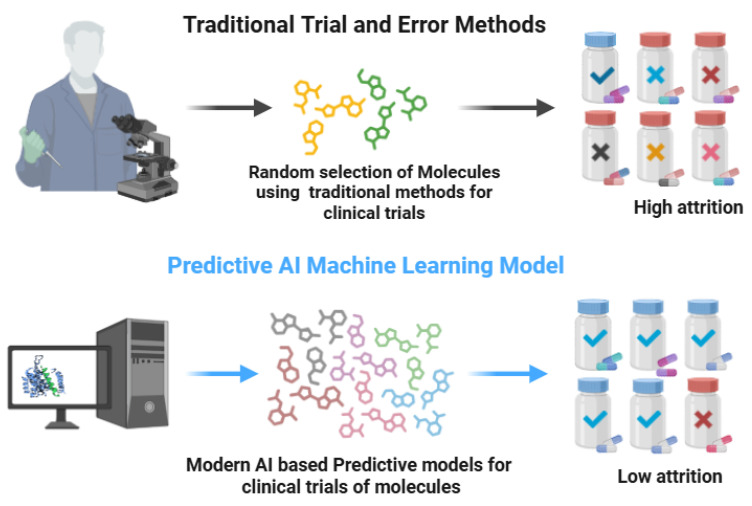
Traditional trial and error vs. modern AI-based discovery approaches (biorender.com, accessed on 28 April 2025). Traditional drug discovery is time-consuming, costly, and has low success rates, whereas the AI-based methods are most effective in the identification of novel drug targets using patients’ genomic data, thus being highly cost-effective with a high success rate.

The traditional trial-and-error approaches for the discovery of new drugs are very multifaceted, time-consuming, and expensive due to the involvement of multiple processes. In the initial target identification phase, researchers use various preliminary processes to find a therapeutic target, like a gene or protein implicated in the pathway of a certain disease [14]. Thereafter, the target is validated through a series of experimental works to link the role of that specific gene or protein with the disease of interest [15]. Then, through a series of experiments, the compound’s genetic library is screened against the target for the identification of possible lead molecules. These compounds are subsequently optimized via medicinal chemistry and pharmacology for their safety, efficacy, and ADME properties [2,16] (Figure 1).

Once the compounds of interest are identified, they are evaluated through different in vitro and in vivo experiments for their preclinical efficacy against the potential targets and their safety through toxicity studies [17]. When considerable safety and efficacy are demonstrated in the preclinical phases, the most effective and potent compounds are subjected to trials in the clinical phase for assessment of their safety and efficacy on humans [18]. The process completion required 10–15 years, with huge costs for the preclinical and clinical phases [11].

### 2.1. Use of AI in Drug Discovery

As discussed earlier, AI is rapidly transforming the field of drug discovery via the acceleration of the processes involved, thus improving efficiency and reducing costs. The AI-based discovery of novel drug molecules involves the process of target identification, de novo design, virtual screening, optimization of the lead molecules, prediction of the pharmacokinetic dynamics, toxicological aspects, and personalized medicine [19].

### 2.2. Target Identification

**Figure 2:**
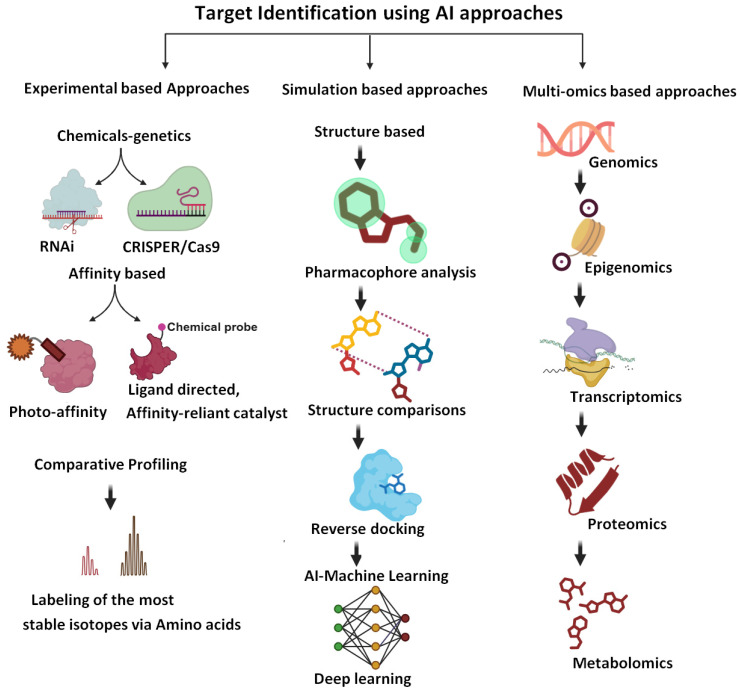
Approaches to the identification of drug targets (biorender.com, accessed on 28 April 2025). The figure summarizes the role of various approaches, including experimental approaches, omics approaches, and molecular simulation-based approaches for the identification of new drug targets.

The identification of drug targets is among the vital and very crucial drug discovery steps, as the preceding steps and their success will depend on appropriate target identification (Figure 2). Using AI-based algorithms, large datasets are analyzed, involving transcriptomic, genomic, and proteomic data, to identify possible targets implicated in the disease pathways or its progression. Subsequent to data analysis, AI identifies genes and proteins with different expressions in disease states, which provides useful information on the identification of molecular mechanisms underlying the biology of the disease. Thus, based on the information of the potential drug, targets can be identified for the development of a modulating agent or a new drug molecule [20].

### 2.3. De Novo Design

The “de novo design” is a process of designing new compounds with definite characteristics via generative models. Using AI tools, new molecules are capable of binding specific targets like enzymes or other proteins, including target receptors. After generating millions of potential molecules, the AI algorithms predict their binding affinity to the target protein, permitting investigators to recognize possible lead compounds. Therefore, the process of new drug discovery is accelerated, and the overall cost is reduced compared to traditional drug discovery approaches [21].

### 2.4. Simulated Screening

The simulated evaluation approach identifies possible hits via the screening of huge libraries of compounds. Using AI tools, large libraries are screened, and the binding affinity of possible compounds against their target proteins is evaluated. The AI algorithms process the structure of the compounds against target proteins, assess their binding affinity, and allow researchers to identify possible lead molecules for further analysis. This approach considerably reduces the synthesis of unsuitable compounds (less affinity and potency), which saves time and valuable resources with better success rates [22].

### 2.5. Lead Optimization

The lead optimization process involves the modification of the physicochemical properties of the molecules for better potency, ADME characteristics, and selectivity. Using AI-based machine learning algorithms, optimization of the lead molecules is achieved. After the analysis of the chemical structure of the molecules, these algorithms predict possible modifications that can increase their selectivity, potency, and ADME properties. This approach can increase the effectiveness and safety of lead compounds, reducing the possibility of unwanted effects associated with the use of these molecules in patients [23].

### 2.6. Predictive Toxicity

Predictive toxicity is the process through which the possible toxicity of the target compounds can be predicted, thus reducing the unnecessary use of experimental animals and increasing the safety of the compounds. Using machine learning algorithms, the possible toxicity of the compounds is predicted. By evaluating the compounds’ chemistry, the AI algorithms foresee their possible toxicity and allow researchers to recognize possible safety issues during the initial phases. Therefore, the unnecessary use of compounds, experimental animals, and overall cost is significantly reduced [24].

### 2.7. Personalized Medicine

The personalized medicine approach involves tailoring treatment plans to individual patients’ needs. Using machine learning algorithms, AI tools create personalized treatment strategies. These algorithms evaluate patients’ data, like clinical, genomic, and transcriptomic data, which predict the utmost effective treatment plan for individual patients. Thus, the patient-related outcomes are considerably abridged by reducing the chances of adverse reactions [25]. The success stories regarding personalized medicine include hypersensitivity reactions, including fever, rashes, and GIT disturbances associated with the use of nucleoside analog reverse transcriptase inhibitors (abacavir) in 5–8% of patients. Genetic analysis revealed the presence of the HLA-B*5701 allele in susceptible patients, with a 70% higher chance of ADRs in these patients. Likewise, the appearance of unwanted skin reactions, including SJS and TEN associated with the use of anti-epileptic agents like carbamazepine and phenytoin, was found to be associated with the presence of the HLA-B*5702 allele in sensitive patients. Subsequently, the FDA recommended screening individuals for the presence of the HLA*5702 allele in patients before taking the medication [26].

## 3. AI Technologies for Drug Discovery

### 3.1. Machine Learning (ML) and Deep Learning (DL)

Important tools, including ML and DL, are transforming the field of drug discovery [27]. ML uses various training algorithms for data analysis to make predictions and conclusions. Using ML-based predictive modeling, data analysis and lead optimization become faster with better success rates. The ML-based deep learning tools exploit neural networks with several layers to evaluate complex data. DL is useful for the analysis or recognition and classification of images, the detection of objects, and natural language processing and generative modeling. The DL models, consisting of convolutional neural networks (CNNs), routinely extract features from huge datasets with the use of manual extraction procedures. The recurrent neural networks (RNNs) are particularly useful for processing sequential data, whereas generative adversarial networks (GANs) create novel data from existing data using various learning features. Using advanced technologies has improved the efficacy and precision of drug discovery processes [27]. Yet, they face some limitations like the complexity of the interpretation process, regulatory frameworks, and quality of the data for accurate predictions [28,29]. Various companies like Atomwise, BenevolentAI, and InSilico Medicine are already leveraging ML and DL in drug discovery. Atomwise has effectively used ML tools for the screening and optimization of lead compounds. InSilico Medicine has utilized DL techniques for designing new molecules against numerous diseases. BenevolentAI has used ML for the identification of new drug targets and the development of personalized medicine [8].

### 3.2. Natural Language Processing (NLP)

Natural language processing (NLP) is based on the interaction of human languages with computers. The NLP contributes significantly to the drug discovery process for literature mining and data extraction, thus enabling researchers to find the most relevant information from large datasets. NLP, after analyzing data sources like text data, patents, and the scientific literature, mines entities including genes, proteins, and diseases for the identification of their relationships. The processes of target identification, compound annotation, and literature reviews have become easier with the use of NLP tools, which save researchers time and costs while aiding in the analysis of optimal sites for drug discovery.

### 3.3. Graph Neural Networks (GNNs)

GNNs are designed for working on data in the form of graphical structures. GNN-type tools can learn node and edge representations, allowing them to foresee different characteristics of nodes and edges [30]. GNNs are effective tools for the estimation of the molecular features of potential drugs and the prediction of their interactions with targets, aiding in the optimization of the molecules. Among the molecular properties, for the stability and bioactivity of the compounds, selectivity and potency assessments are performed. Benefits associated with GNNs include the accuracy, speed, and efficiency of the drug screening processes. Yet these AI tools are associated with issues with interpretation, the quality of the data used, and the regulatory frameworks [31].

### 3.4. The TxGNN (Transformer-Based Graph Neural Network)

The TxGNN is one of the deep learning models that utilize a thorough knowledge graph for the prediction of possible drug candidates for new drug discovery and repurposing of old drugs [32]. The AI tool, by utilizing a massive information graph related to drugs and diseases, embeds them into a latent space and improves the demonstration of the knowledge graph’s structure. The TxGNN’s design comprises a metric learning module that allows the transfer of information from well-explained diseases to those having limited data, thus allowing the production of real-world forecasts against various disorders [33]. Moreover, the explainer of this model offers clear validations regarding the forecasts made via multi-hop paths connecting drugs to diseases. This tool has provided exemplary accuracy regarding the prediction of drug indications as well as contraindications in comparison to the available methods. The capacity of TxGNN to provide reasonable forecast upsurges is supported by its results, making it an important tool for decision-making in clinical settings. By coupling the current uses of drugs with TxGNN-based predictions for new uses, the discovery of modern therapeutics against various diseases can be accelerated [34].

### 3.5. The Role of AlphaFold-2

This revolutionary AI model, originally established by Google DeepMind, is capable of predicting the 3D structures of proteins with extraordinary precision [35]. It has enabled researchers to explore the complex protein interactions and their function, which has transformed the process of new drug discoveries. This tool helps investigators identify the drug targets to design novel biologics and small molecules [36]. After precise prediction of the protein’s structure, this AI tool provides in-depth insights into structure-based drug design, allows molecules to advance the design of personalized medicine, and enhances the safety and efficacy of novel therapeutics. Thus, the impact of this AI invention encompasses the understanding of the underlying mechanisms of various diseases, as well as the identification of potential targets against neurodegenerative disorders, and can transform the field of drug discovery [37].

### 3.6. The Logica AI Platform

Logica is another cutting-edge AI platform that exploits ML and data analytics to initiate business revelations and problem-solving [38]. After an analysis of the complicated databases, this AI tool recognizes patterns and creates proactive models; it also allows businesses to identify unseen tendencies, estimate forthcoming products, and make informed decisions [38]. Having broad applications across numerous industries—such as healthcare, product marketing, and finance—this AI tool can perform industrialized, tedious tasks and help optimize workflow with enhanced productivity [39]. Moreover, this AI tool has the ability to analyze data and model predictions, as well as an automation capacity to enhance its utility for industries seeking better AI-based innovation and growth, hence the increased significance of this AI tool [39].

### 3.7. NeuroCADR

NeuroCADR is an algorithmic process that has achieved considerable significance in the identification of new uses for the marketed drugs against CNS disorders [40]. Using hybrid approaches, this tool can speed up cost-effective drug discovery compared to classical drug development approaches. By optimizing the sophisticated computational techniques for the analysis of multifaceted neurological data, this tool can help in the discovery of new neuroprotective drugs. NeuroCADR can predict the structures of proteins and their interactions via cutting-edge algorithms to identify molecules capable of targeting the proteins of interest as potential therapeutics against neurological diseases. It also has the capacity to generate predictive models for better drug safety and efficacy and fewer side effects [40,41].

### 3.8. Isomorphic Labs

Another AI tool is Isomorphic Labs, which was developed by a company operating in the UK and is centered towards the discovery of new drugs. The founder of this tool, Demis Hassabis—who is the co-founder and chief executive of DeepMind—utilizes innovative AI expertise to accelerate novel drug discovery. By using ML algorithms, the company scrutinizes multifaceted biological data for the identification of drug targets and the design of novel compounds. With an AI influence, Isomorphic Labs intends to revolutionize the process of drug discovery by increasing the efficiency of production and safety and the efficacy of drugs [42].

### 3.9. RoseTTAFold

RoseTTAFold is a highly efficient, AI-driven tool capable of predicting proteins’ 3D structures with exceptional accuracy. Because of its accurate analysis of the proteins’ structure and their interactions, this tool can identify novel drug targets and help in designing innovative therapeutic modalities. By analyzing interactions between small molecules and their biological targets, this tool has made the discovery process for various diseases—including neurological disorders—more efficient [43].

## 4. Alzheimer’s Disease (AD)

**Figure 3:**
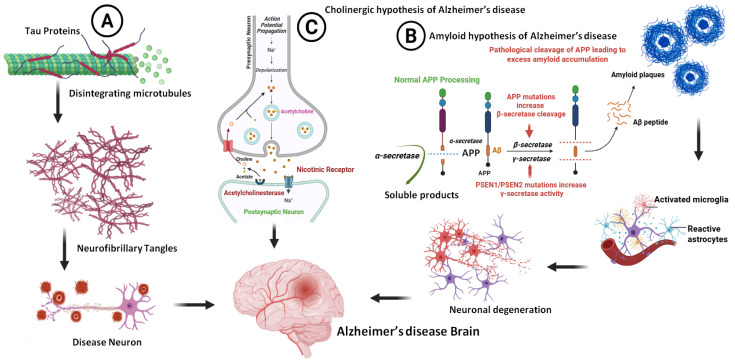
Pathological aspects of AD (**A**), the formation of NFTS, (**B**), the formation of Aβ, and (**C**) the cholinergic hypothesis and role of cholinesterase inhibitors (biorender.com, accessed on 28 April 2025).

AD is a degenerative disease of the nervous system among the elderly population, which drastically affects patients’ thinking, memory, and behavior [44]. Among the key features of AD are gradual cognitive decline, memory issues, agitation, and behavioral changes, including mood swings and anxiety [45]. It is a progressive disease passing through various stages, including preclinical to mild cognitive impairments, followed by early-stage, moderate, and late-stage AD, with a gradual yet significant increase in cognitive defects, leading to a complete reliance on caregivers [46]. Causes of AD and risk factors often revolve around genetics, age-associated decline, sedentary lifestyles, the lack of physical activities, and social limitations, which are all implicated in the progression of AD [47]. Pathophysiological aspects of AD include the accumulation of Aβ, NFTs, cholinergic scarcity, excessive free radical formation, and inflammatory processes leading to neuronal damage and dysfunction [48] (Figure 3). Due to the loss of cholinergic neurons, there is an obvious decline in acetylcholine (Ach) levels, which plays a role in memory. Subsequently, efforts are directed to use agents that can inhibit the enzyme involved in Ach metabolism to augment its neurological activity [49]. In this regard, inhibitors of the enzymes cholinesterases (AChE and BChE) are among the most successful strategies, and several drugs have been approved for clinical use. Other agents include glutamatergic modulators (memantine) and anti-inflammatory and antioxidant agents, which are also used concomitantly [50,51,52]. Various other therapies aimed at counteracting amyloid production or its clearance from the body are in different stages of clinical trials [53]. Unfortunately, the development of anti-AD therapeutic agents is very limited, as only few agents capable of relieving the symptomatology have been developed, and the search for more effective drugs is underway.

### 4.1. Role of AI in Disease Detection, Disease Assessment, and Drug Discovery

The AI-based resources can be effectively applied to data sources including biomarkers, genetic information, medical images, clinical records, and the EEG signals from AD patients. Medical information from these sources, using various techniques such as PET, SPECT, fMRI, computed tomography (CT), and MRI—when coupled with AI algorithms—considerably contributes to assessing the level of brain pathology and the stages of the disease. Moreover, these tools have high diagnostic accuracy, which might support the early detection of AD [54]. By using the AI tools, complex patterns in medical images can be detected, and the quantitative assessments of the radiological data can be performed quickly and more effectively. Thus, the accuracy of the analysis and the outcomes is enhanced. So, the integration of AI in the radiological evaluation of diseases has facilitated faster, economical, and consistent analyses of the images [55].

Various serum- and CSF-based biological samples can be effectively analyzed for the presence of protein markers via AI-based diagnostic tools. Through an analysis of AD-indicative protein expression—including Aβ, tau, NFTs, and other peptides—the machine learning algorithms provide information about abnormal expressions. Researchers have reported the exploration of iron dysregulation in AD patients using an AI-based analysis of patient data. Their findings indicate that iron dysregulation interacts with AD biomarkers, possibly affecting the progression of dementia [56]. During the course of neurodegeneration in AD patients, there is a progressive decline in articulation and language. Using AI-based computational speech processing tools, these changes can be predicted and diagnosed as initial diagnostic tools. A study explored the possibility of automatic speech analysis as a diagnostic tool for mild cognitive impairment (MCI) during early-stage AD [57]. Another study, which used AD patients vs. healthy controls, implemented short cognitive vocal tasks for the analysis of speech as a diagnostic tool [58]. Another study utilized vocal indicators from AD patients and normal individuals using speech signal processing techniques for the detection of AD in terms of MCI. Machine learning methods were successfully used for the creation of automatic classifiers, as they have exceptional accuracy, and were rated as 80% for AD vs. MCI, 87% for AD vs. controls, and 79% for MCI vs. controls. These findings validate the potential use of automatic audio analysis in the assessment of AD and MCI [59,60]. Yet, the field faces limitations, such as disconnection among the clinical uses and aims, inadequate comparability of the findings, and low standardization, which highlight the areas of future improvement and further modifications.

#### 4.1.1. Early-Stage Diagnosis of the Disease

The diagnosis of AD in the early stages has a profound clinical impact, allowing well-timed interventions and possibly better therapeutic and management outcomes. Numerous imaging techniques—like MRI, amyloid-PET, FDG-PET, and tau-PET—coupled with AI-based diagnostic tools have tremendous advantages in terms of the most appropriate diagnosis and prognostication during the early stages of AD, like MCI or the preclinical stages. Broadly, these imaging techniques either help in the detection of neuronal damage or help in the identification of amyloid positivity. Various studies have highlighted the early pathophysiological changes in AD, including glucose metabolism and the deposition of Aβ in individuals with normal cognitive performance using the FDG-PET analysis [61]. Likewise, studies revealed the link between hypo-metabolism and the deposition of Aβ among individuals with normal cognitive performance [61]. Researchers used machine learning tools for AD prediction using multimodal connectome data from one hundred and ten healthy controls and preclinical AD individuals. Via the integration of morphological, anatomical, and functional networks, these models have attained an accuracy of 88.73% in the identification of AD patients in the preclinical stages. This signifies the potential of multimodal imaging and machine learning in early AD detection and prediction [62,63].

#### 4.1.2. Predicting the Progress of the Disease (MCI to AD)

Researchers have tried to predict the progression and length of time taken for the conversion of MCI until the appearance of full symptoms of AD [64]. Information, including cortical thickness and brain connection network features obtained from structural MRI and resting-state fMRI (rs-fMRI), can help differentiate among patients with MCI converters and those with MCI non-converters [65]. The results advocate for the idea that the converted sensitivity brain regions of the two patient groups (MCI non-converters vs. MCI converters) and the same group of patients (MCI converters vs. AD) may vary, allowing a precise estimation of MCI conversion to AD. The time required for MCI to fully develop into AD in individuals is highly complex due to variable progress rates among individuals. Yet, the AI-based prediction models using the influencing factors can help to better understand the disease progression [66]. A group of researchers presented a new method to subdivide the MCI patients on the basis of their conversion time [67]. Their findings revealed that the stratified classifiers can foresee translation from baseline for three years with a precision of seventy-four percent, exhibiting great specificity (eighty-four percent) but low sensitivity (sixty-four percent). To be therapeutically valuable, the lasting prediction’s sensitivity must be upgraded to exploit the benefits of potential neuroprotective treatments.

#### 4.1.3. Stages and Classification of AD

Despite widespread studies on cognitive analysis, the majority of the research is directed towards the detection of cognitive impairments. By using medical images, AI can identify various stages of the disease. For instance, by using 6400 MRI images, researchers categorized AD into four stages, including moderate dementia, non-dementia, very mild dementia, and mild dementia. While utilizing the CNN method, they precisely anticipated and divided the stages of AD with 97.19% accuracy [68]. Likewise, another group of researchers used resting-state fMRI data and Gaussian process classification to differentiate normal controls from patients with mild cognitive impairments and AD [69]. Multivariate statistical machine learning techniques were used to evaluate the efficacy of the patient’s classification on the basis of brain functional connectivity patterns [69]. The accuracy rate for this model in differentiating the amnesic MCI from healthy controls was seventy-five percent, whereas this accuracy was seventy-nine percent for amnesic MCI vs. AD patients, which clearly validates the potential role of AI in the AD prediction and its classification. Using a multi-feature kernel discriminant dictionary learning approach, researchers grouped the AD into different stages, including no cognitive impairment, MCI, and AD [70]. The classification accuracies based on this method were no cognitive impairment vs. AD (98.18%), no cognitive impairment vs. MCI (78.50%), and MCI vs. AD (74.47%), which clearly highlights the role of AI tools in the accurate prediction of the stages of cognitive impairment and its progression towards AD [70].

#### 4.1.4. Blood-Based AD Biomarkers

The analysis of blood-based AD biomarkers received considerable attention due to their potential applications in the diagnosis, monitoring, and treatment of the disease. Several markers for key pathological targets like Aβ [71], tau proteins [72], inflammation markers [73], lipids, neurofilament light chains [74], and metabolic markers have been identified as key diagnostic markers [75]. In comparison to CSF biomarkers or imaging modalities, blood-based markers are easily accessible from the plasma or serum with minimally invasive interventions. The blood-based markers have been used to differentiate between healthy individuals and those with AD, as well as to evaluate the progression of the disease, cognitive decline, and response to therapeutic agents [75,76]. For example, elevated NFL levels have been found to be a key marker in the blood of AD patients [77]. Likewise, changes in lipid metabolism have been determined to be an important pathophysiological indicator for AD [78]. Researchers have used machine learning and AI tools for the investigation of plasma AD metabolites as a possible diagnostic tool. LC-MS coupled with statistical approaches and proton NMR can help in the identification of several metabolites that can differentiate between MCI, AD, and healthy individuals [79,80].

#### 4.1.5. Urine-Based Biomarkers

The urine-based biomarkers for mild-to-moderate AD can also be analyzed using AI tools. The efficacy of a quantitative metabolomics technique coupled with MS and proton NMR for the analysis of urine-based metabolites is reported [81]. Investigators utilized LASSO and CFS approaches for the creation of biomarker panels, which were measured via logistic regression models and SVM. Eleven AD metabolites were identified in the study, including tryptophan, malonate, cytosine, hippuric acid, N-oxide (trimethylamine, 2-ketoisovalerate, 2- and 3-hydroxyisovalerate, and urocanate), guanidinoacetate, and glucose. These biomarkers established the potential for diagnosing AD phases, achieving 81% precision, 75% specificity, and 76% sensitivity [81]. Using AI involves the analysis of AD biomarkers in urine for the confirmation of AD stages. AI tools like machine learning and DL can be utilized for the identification of patterns in urine metabolite data, to develop predictive models for the diagnosis of AD, and to validate biomarker panels. Urine-based markers like amino acids, malonate, trimethylamine, and other compounds like hippuric acid can be analyzed via AI-based tools. By leveraging AI-powered analysis, urine-based biomarker detection may offer a non-invasive and economical approach for AD diagnosis and monitoring, potentially allowing earlier intervention and more effective disease management.

#### 4.1.6. MRI-Based Biomarkers

MRI data can be evaluated by machine learning algorithms to recognize elusive alterations in the brain structure, revealing AD characteristics. In a neuroimaging project (ADNI), researchers used an upgraded machine learning algorithm to analyze MRI data from more than 500 individuals, consisting of 162 healthy controls, 134 MCI non-converters, 76 MCI converters, and 137 AD patients [82]. The study discovered biomarkers linked with specific brain regions, such as the hippocampus and entorhinal cortex, which are crucial in the pathophysiology of AD. These biomarkers allowed classification accuracies of 66% for MCI converters vs. non-converters, 72% for MCI converters vs. healthy controls, and 76% for AD vs. healthy controls. This AI approach has the capacity to improve the early analysis and management of AD by identifying elusive variations in brain structure [82].

## 5. AI-Driven Discovery of Drugs Against AD and Neurodegenerative Diseases

The use of AI has considerably accelerated the process of drug discovery against various diseases, including neurological disorders. By analyzing huge amounts of data, AI-based tools can simulate the effects of potential drugs, thus helping in the discovery of novel drugs. AI tools use the processes of target identification, lead generation, optimization, and then preclinical development. In the initial step of the target identification process, these AI tools use several datasets to identify patterns that assist in the recognition of molecular events underlying diseases and therapeutic actions. The AI algorithms also systematize and improve novel drug design processes, both scoring functions, and QSAR models in simulation study pipelines. Moreover, AI methods utilize the physicochemical characteristics of the molecules in the preclinical phases to appraise the ADME of the compounds for lead identification, which accelerates the process of drug discovery.

The AI tools also contribute towards anti-AD drug discovery by examining huge quantities of data to recognize potential therapeutic targets and design and optimize new compounds. They also help in the prediction of the efficacy and toxicity of these compounds and integrate large datasets to identify patterns and insights. All of these processes accelerate the discovery of anti-AD drugs by increasing efficiency, reducing costs, enhancing accuracy, and facilitating personalized medicine. By leveraging machine learning algorithms and predictive modeling, AI has the potential to revolutionize the field of AD research, aiding the development of effective treatments and improving patient outcomes.

## 6. Molecular Docking as a Screening Tool for Anti-AD Drugs: BACE1 and Cholinesterase Inhibitors as Examples

**Figure 4:**
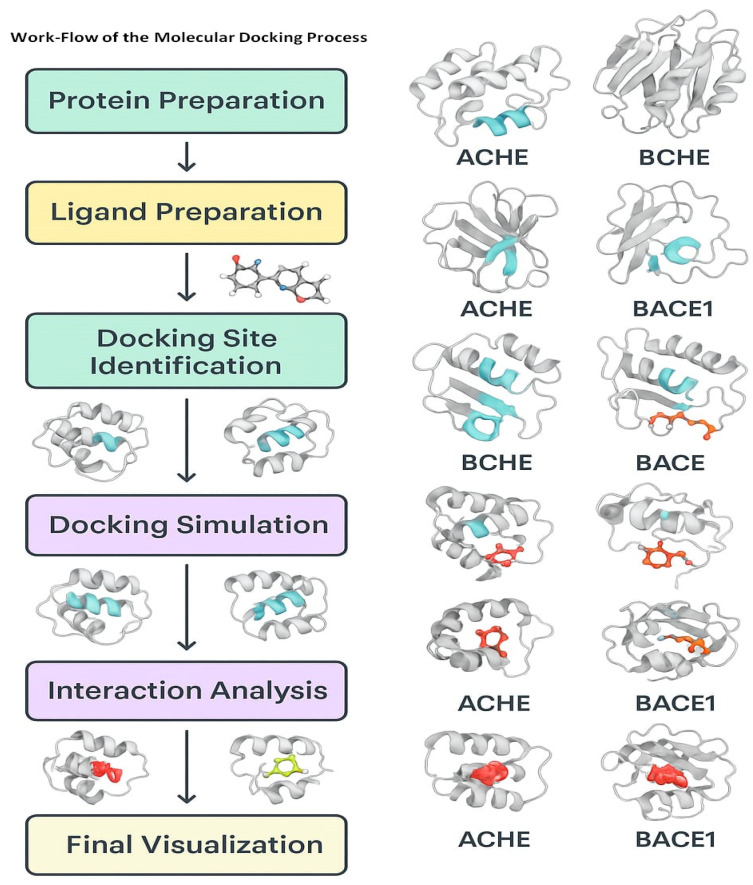
Workflow of the simulation-based analysis of the potential molecules against the enzyme targets of AD (images were generated using MOE (Moe2018) docking software).

Molecular docking is an important computational approach applied to the process of drug discovery, which evaluates the interaction of ligands with proteins and predicts the binding mode and affinity of these molecules with their protein target [83] (Figure 4). By evaluating the binding affinity of molecules with their target proteins using docking approaches, the identification of potential lead molecules among the large group of molecules becomes easy. By evaluating large libraries of compounds quickly and more efficiently, this approach will revolutionize the field of drug discovery [84,85].

Among the key steps in the molecular docking studies are the preparation of the target, the preparation of the ligand, molecular docking, and their interaction scoring [86,87]. During the target preparation step, the 3D structure of the target protein is prepared, followed by the preparation of the binding site [88]. Ligand preparation consists of creating a 3D structure of the potential drug molecule. Subsequently, docking includes the use of a docking algorithm for the prediction of the binding mode of the ligand molecules with the target protein [89]. Finally, the binding affinity of the ligand and protein is predicted from the scoring functions.

Among the key advantages of the molecular docking approach for drug discovery are high-throughput screening, precise predictions, and very low false-positive rates. By screening large libraries of potential compounds, the docking tools can precisely predict the binding mode and affinity of small molecules, reducing the number of false positives and saving time and resources [83]. In spite of its numerous advantages, the docking approach faces several limitations and challenges. For instance, the precision of scoring functions may vary considerably and usually depend on the specific type of algorithm used. Moreover, the flexibility of proteins usually makes it very hard to predict the binding mode and affinity of small molecules. Moreover, molecular docking faces difficulties in predicting the appropriate binding mode of ligands for highly complex protein–ligand systems [83].

## 7. AI-Driven Drug Discovery Against Parkinson’s Disease

Parkinsonism is another complex neurodegenerative disease associated with motor dysfunctions including muscular rigidity, tremors, and bradykinesia. Current therapeutic agents have limited symptomatic efficacy, necessitating the discovery of more effective disease-modifying agents. In comparison to the traditional methods, AI-based approaches have emerged as powerful tools offering extraordinary prospects for accelerating the development of novel drugs against PD. The AI-based methods using machine learning algorithms are effective in quickly screening a huge number of compound libraries and identifying molecules capable of modulating vital PD-related targets, including α-synuclein, GCase, and LRRK2 [6]. They also help to analyze large datasets and provide new insights into the pathological aspects of the PD markers, thus helping with better diagnoses and drug discoveries [11]. Research studies demonstrated the use of AI for the identification of molecules having inhibitory potentials against α-synuclein aggregation, which is a vital target of PD [9]. It was also effective in designing new compounds that target the protein kinase LRRK2, another pathological target implicated in PD pathogenesis [90].

## 8. AI-Driven Drug Discovery for Multiple Sclerosis (MS)

Another neurological disorder is MS, which is an autoimmune-linked degenerative disorder causing neuronal demyelination, inflammation, and neurodegeneration. Due to the unavailability of effective therapeutics against MS, there is a dire need for the discovery of more effective and safer therapeutic remedies. AI-driven approaches have been effectively used for the identification of therapeutic targets as well as potential drug leads. By screening the compound libraries, the AI-based algorithms help in the identification of drug candidates and are effective in modulating key MS-related targets, such as S1PRs and immune cells [6]. A group of researchers used AI tools for the identification of small molecules with inhibitory potentials against S1PRs, a vital target in MS pathogenesis [9]. AI is also in use for designing new molecules that target immune cells (T and B cells), which are associated with inflammation-mediated demyelination in MS patients.

## 9. The Role of AI in Drug Discovery for Epilepsy

Epilepsy is a highly prevalent and complicated disorder of the brain that is associated with recurrent episodes of seizures and other complications. Based on the pathophysiological aspects of the disease, several therapeutic remedies have been devised. But unfortunately, they have been shown to have limited efficacy and side effects. AI-based innovative approaches are effective in identifying novel therapeutic targets against the disease and offer extraordinary opportunities for the discovery of anti-epileptic drugs. By rapidly evaluating the large libraries of natural and synthetic compounds, ML algorithms can locate molecules of interest that can modulate epilepsy targets, including neurotransmitter receptors and ion channels [6]. After evaluation of the large datasets, the AI can reveal new information about the pathological targets of the disease and help in the identification of biological markers for better diagnoses, disease prevention, and disease response to drugs.

Research studies revealed the contribution of AI-based approaches for the identification of novel anti-epileptic drugs. Studies using AI tools have identified small molecules with GABA-receptor-potential modulators, which regulate the excitability of neurons and contribute to the activity of seizures [9]. Moreover, AI helped in the identification of molecules targeting voltage-gated sodium channels for the management of epilepsy [91]. The integration of AI tools in the drug discovery for epilepsy can accelerate the process of target identification, aid in lead optimization, and improve predictive models which can help in the disease progression and response to therapeutics.

## 10. The Role of AI in Drug Discovery for ALS

Amyotrophic lateral sclerosis (ALS) is an irreversible degenerative disease of the neuronal cells in the brain and spinal cord. Weakness and gradual decline in the nerve cells lead to poor control over the muscular activities, causing paralysis of the muscles and eventual death of the nerve cells. ALS is associated with motor neuron degeneration, which controls the voluntary movements of the muscles. With very limited drugs available for its management, ALS has no complete treatment and can have fatal consequences if not handled properly [92].

The integration of AI into the drug discovery process contributes significantly by using ML algorithms, which help to identify novel therapeutic targets and more effective molecules with better safety and efficacy profiles [93]. By evaluating the relevant complex biological omics data, these AI tools provide new insights into the disease’s pathology, including the structure of the proteins, and can effectively simulate the interactions of possible lead molecules against the targets [94]. These interactions help in the design of new modulators of the biomolecules associated with the pathology of ALS. Moreover, evaluation of the patients’ relevant data and outcomes of the clinical trials can support the design of personalized medicine against these complex diseases [95].

## 11. Benefits and Challenges Associated with AI-Based Drug Discovery

The incorporation of AI tools has transformed the process of drug discovery and offers numerous benefits. By rapidly analyzing the huge amounts of data, AI tools can efficiently identify drug target candidates and predict their toxicity and efficacy [6]. This not only accelerates the process of drug discovery but also tremendously improves the accuracy and efficacy of the process and reduces the cost significantly [90]. Moreover, by tailoring treatment to the individual needs of patients based on their genetic analysis and medical history, AI has made the personalization of medicine extremely effective [96]. AI-based analysis can provide new insights to identify new drug targets after analysis of the complex biological systems and pathways involved in the physiological and pathological processes in the body [9].

The AI-based discovery processes also face numerous challenges. Among these, the quality of data is of high clinical significance in the context of products [97]. These tools need data inputs of high quality with diversity and relevancy for more accurate predictions and outcomes. The lack of interpretability is another problem for AI-directed decisions and predictions [98]. Moreover, regulatory frameworks must extend to AI-based drug discovery, which ensures the drug’s safety and efficacy [99]. AI-based models can perpetuate biases and variability in data, which affect the quality of predictions and integration with the existing processes, and necessitate appropriate infrastructure and collaboration with other researchers [100]. The emergence of various companies, including InSilico Medicine, Atomwise, BenevolentAI, and Berg Health, has modified AI to recognize various molecules as targets for diseases like cancer, Ebola, and fibrosis [9]. These cases highlight AI’s ability to speed up the discovery and improve efficiency and innovation in the drug discovery process. To effusively leverage the role of AI in drug discovery, it is important to evaluate the associated challenges and overcome the limitations. The use of hybrid approaches that combine AI tools with traditional approaches for designing explainable AI models can offer transparent and interpretable results and encourage collaboration with pharmaceutical industries for data sharing. After addressing these challenges and experiencing the benefits of AI, the pharmaceutical industry can create new opportunities for innovative treatments with better patient outcomes.

The integration of AI tools with healthcare is associated with numerous ethical concerns, which include issues related to the privacy of patients’ data and the use of informed consent, as well as the absence of transparency in making AI-based decisions [101,102]. To cope with these challenges, researchers are required to implement robust data protection procedures, including encryption and protected data storage, which ensure better compliance with relevant regulations such as HIPAA and GDPR [103]. For the mitigation of bias in using AI tools, the use of different and representative datasets, data quality control, and bias detection methods is required [104,105].

## 12. Future Directions and Opportunities

The inclusion of AI-driven approaches has revolutionized the field of drug discovery, with numerous emerging trends and opportunities on the horizon. The integration of AI with evolving technologies like CRISPR gene editing, single-cell analysis, and synthetic biology has emerged as avenues for new discoveries. This convergence of technologies has the potential to reveal new targets regarding the prevention and treatment of diseases and speed up the discovery of novel therapeutics. Moreover, the emerging role of AI-driven personalized medicine will help in tailoring treatment to individual patients on the basis of their unique genetic make-up and medical histories.

Another important aspect of AI inclusion in drug discovery is its role in predictive modeling. AI can help researchers with earlier and more accurate diagnoses of diseases and gauge the disease progression and response to therapeutic agents. Collaborative projects and data sharing with pharmaceutical industries will accelerate the process of innovation, especially drug discovery. In this regard, the development of explainable AI models that offer transparent and interpretable results might be required to augment the trust and stability in AI-driven decision-making. As AI continues to transform the field of drug discovery, we can expect to see better patient outcomes, improved efficiency, and enhanced innovation. More useful and targeted therapies will gradually become available, and discovery and development processes will become more streamlined. The discovery of novel targets for drug therapy, new markers, and compounds effective against multiple biological targets of the disease can reveal better therapeutic prospects against serious diseases. Subsequent to the implementation of these prospects, drug development industries can benefit from the potential of AI and achieve significant progress in the discovery of cost-effective molecules against neurodegenerative diseases, including AD.

## Data Availability

Any data related to this manuscript is available to researchers upon request.
